# Title Changes in Plasma Levels of Selected Matrix Metalloproteinases (MMPs) Enzymes in Patients with Osgood–Schlatter Disease (OSD)

**DOI:** 10.3390/jcm13195655

**Published:** 2024-09-24

**Authors:** Monika Kulesza, Tomasz Guszczyn, Aleksandra Kicman, Sławomir Ławicki

**Affiliations:** 1 Department of Population Medicine and Lifestyle Diseases Prevention, Medical University of Bialystok, 15-269 Bialystok, Poland; monika.kulesza@sd.umb.edu.pl; 2 Department of Pediatric Orthopaedics and Traumatology, Medical University of Bialystok, 15-274 Bialystok, Poland; tomasz.guszczyn@icloud.com; 3 Department of Aesthetic Medicine, Medical University of Bialystok, 15-267 Bialystok, Poland; olakicman@gmail.com

**Keywords:** matrix metalloproteinases, MMP-26, MMP-7, MMP-9, Osgood–Schlatter disease, sterile bone necrosis

## Abstract

**Background**: Osgood–Schlatter disease (OSD) belongs to the group of sterile bone necrosis and mainly affects athletically active children. The pathogenesis of OSD is currently not fully understood, so the purpose of this study was to evaluate the concentrations of selected matrix metalloproteinases (MMPs)—MMP-2, MMP-3, MMP-7, MMP-9, MMP-10 and MMP-26 in patients diagnosed with OSD compared to patients with diseases other than sterile bone necrosis **Methods**: The study group included 140 patients with OSD, while the control group contained 100 patients with knee pain unrelated to sterile bone necrosis. The MMPs tested were determined by an enzyme-linked immunosorbent assay in plasma. **Results**: Patients with OSD had higher concentrations of MMP-2 and MMP-9 compared to the control group. The concentrations of MMP-7, MMP-10 and MMP-26 were lower in affected children. High values of diagnostic parameters—diagnostic accuracy (AC), sensitivity (SE), specificity (SP) and area under curve (AUC)—were obtained for MMP-7, MMP-9 and MMP-26. **Conclusions**: The collected results convince that MMP-7, MMP-9 and MMP-26 can be consider as a differential ancillary test between OSD and other knee pain and may be involved in the pathogenesis of this condition.

## 1. Introduction

Osgood–Schlatter disease (OSD) belongs to the group of sterile bone necroses [1]. It usually affects children and adolescents between the ages of 8 and 15 who are active in sports and manifests as pain and tenderness in the tibial tuberosity region [1,2,3,4]. The clinical diagnosis of Osgood–Schlatter disease is mainly based on clinical history and imaging studies [3,5]. OSD is mostly treated conservatively with ice packs, immobilization of the limb, rehabilitation or administration of nonsteroidal anti-inflammatory drugs (NSAIDs) [1,3,5]. The disease is self-limiting in most cases; however, the process can take up to two years [3,4]. Although acute or chronic strains during sports activities causes inflammation of the attachment of the patellar tendon to the tibial tuberosity, the etiology and etiopathogenesis of OSD is not fully understood [3,6].

Extracellular matrix metalloproteinases (MMPs) are a group of zinc ion-dependent enzymes. They are divided into six groups depending on their substrate specificity, i.e.,: gelatinases, collagenases, stromelysins, matrilysins, membrane-type metalloproteinases and others [7,8,9,10]. The activity of MMPs is crucial in maintaining tissue allostasis, mainly through the degradation of extracellular matrix (ECM, eng. extracellular matrix) components [7,10]. Physiologically, MMPs are maintained in a state of equilibrium; the dysregulation of their activity has been linked to the development of a number of diseases such as cardiovascular, nervous, excretory and respiratory diseases. A particular role for these enzymes has been attributed to the formation and progression of cancer [7,10,11].

MMPs are expressed in individual bone cells such as osteoblasts, osteoclasts and chondrocytes [12,13]. Although the exact role of these enzymes within bone has not been thoroughly investigated, MMPs have been shown to be associated with processes such as cell adhesion, bone tissue remodeling and also bone cell proliferation differentiation and apoptosis [9,12,14]. The available literature data indicate that regular physical activity can affect the level of MMPs. The level of MMPs also varies with training intensity. Recreational exercise has a moderate effect on the concentrations of MMP-2 [15] and MMP-9 [15], where an increase in these metalloproteinases is observed [16]. Resistance training was associated with an increase in MMP-9, while with callisthenic training, only a transient increase is found [17]. Resistance training had no effect on MMP-3 concentrations [17].

Importantly, excessive MMPs activity is associated with the development of bone diseases such as osteoporosis, degenerative spine disease and bone tumors [12,18,19]. An increase in the concentrations of individual MMPs is observed in the course of bone disease, so they can be considered as potential markers of osteoporotic diseases [12,20,21]. Of particular note are MMPs such as MMP-2, MMP-3, MMP-7, MMP-9, MMP-10 and MMP-26. These enzymes are directly involved in the metabolism of collagen and other connective tissue structures and bone elements. Their activity has been shown to be closely associated not only with ossification, but also with bone regeneration and also with many pathological processes within the skeletal system [12].

The pathogenesis of OSD has not been fully elucidated at present. Given that MMPs are involved in both physiological and pathological processes within bone it is highly likely that they also mediate the formation of this condition. Therefore, the aim of the present study was to evaluate the concentrations of selected MMPs in patients with OSD compared to patients with mild musculoskeletal injuries and to demonstrate potential differences in the levels of these enzymes that could indicate a role for MMPs in the pathogenesis of OSD.

## 2. Materials and Methods

### 2.1. Patient Characteristics

The study included 140 patients—71 girls and 69 boys who presented with symptoms of pain in the knee area to the Department of Orthopedics and Traumatology of the Children’s Clinical Hospital in Bialystok. The diagnosis of Osgood–Schlatter disease in patients was based on their clinical history and radiographic findings. Clinical examinations identified pain, swelling and/or hypertrophy in the tibial tuberosity area. X-ray images revealed an enlarged tibial tuberosity contour and/or loose bone fragments at the patellar ligament attachment site. Patients with a history of knee trauma or other knee conditions were excluded.

The control group (CG) consisted of 100 patients (38 girls, 62 boys), aged 12 to 15 (median: 13), who presented to the Department of Orthopedics and Traumatology of the Children’s Clinical Hospital in Bialystok for due to knee pain other than OSD. The characteristics of the groups are shown in Table 1.

The study was conducted in accordance with the Declaration of Helsinki. The protocol was approved by the local Ethics Committee of the Medical University of Bialystok (committee approval numbers: APK.002.580.2021 (approval date 16 December 2021). The participants in the study were minors; hence, after consulting their parents/legal guardians and obtaining written consent, the children were qualified to participate in the study.

### 2.2. Biochemical Analysis

The main study material was plasma. For this target, blood was collected from patients for anticoagulant—lithium heparin. Blood samples were centrifuged at 3000 rpm for 10 min at room temperature. The blood was centrifuged once. In this way, a supernatant was obtained, in which the top layer was plasma. Plasma was separated from the supernatant by using an automatic pipette. Holding the test tube at a suitable angle, using a disposable tip of automatic pipette, the plasma was extracted without disturbing the layers formed during centrifugation and without allowing the plasma to mix with morphotic elements. Plasma then was stored at −81 °C until testing day.

Determination of selected metalloproteinases was carried out by enzyme-linked immunosorbent assay (ELISA). The assay used kits from R&D Systems (MMP-2: Human MMP-2 DuoSet ELISA, Cat. Cat. no. DY902; MMP-3: Human Total MMP-3 DuoSet ELISA, Cat. no. DY513, Human Total MMP-7 DuoSet ELISA, Cat. No. DY907, Human Total MMP-9 DuoSet ELISA, Cat. No. DY911, Human Total MMP-10 DuoSet ELISA, Cat. No. DY910; Minneapolis, MN, USA) and Abbkine (MMP-26: Human Matrix metalloproteinase-26 (MMP26) ELISA Kit, Cat. Cat. no. KTE61590, Georgia, GA, USA). The test procedure was performed according to the manufacturer’s recommendations included with each kit.

Standards and patients were applied to the plate in duplicate. The precision of the sets was determined by the manufacturer—MMP-2: 3.8% (intra-assay), 6.6% (inter-assay); MMP-3: 6.1% (intra-assay), 7% (inter-assay); MMP-7: 3.7% (intra-assay), 4.1% (inter-assay); MMP-9: 1.9% (intra-assay), 7.8% (inter-assay); MMP-10: 3.7% (intra-assay), 4.1% (inter-assay); and MMP-26: <9% (intra-assay), <11% (inter-assay). Plates were read using a wavelength of 450 nm and a correction set at 540 nm with use a microplate reader from BIOKOM.

### 2.3. Statistical Analysis

Statistical analysis was performed using the statistical language R (version 4.3.3; R Core Team, 2024) on Windows 11 Pro 64 bit (complication 22000), and figures were produced using GraphPad Prism 5 Software (GraphPad Software, La Jolla, CA, USA). Assessment of the significance of intergroup differences for numerical variables that did not meet the assumption of normality of distribution was performed using the nonparametric Wilcoxon rank sum test.

Independence between the two variable categories was assessed using Pearson’s Chi-square test. Using the ROC curve, an analysis of the reliability and diagnostic power of the tests was carried out with the determination of the optimal cutoff point for MMP-2 (≥0.092 pg/mL), MMP-3 (≥1.81 ng/mL), MMP-7 (≤0.47 ng/mL), MMP-9 (≥132.10 ng/mL), MMP-10 (≤0.47 ng/mL) and MMP-26 (≤7.83 ng/mL). To assess the significance of differences in the discriminatory abilities of different models, we used the AUCs calculated by the DeLong statistical test.

## 3. Results

### 3.1. Plasma Concentrations of MMP-2, MMP-3, MMP-7, MMP-9, MMP-10 and MMP-26 in Patients with OSD and Control Group (CG)

**Figure 1:**
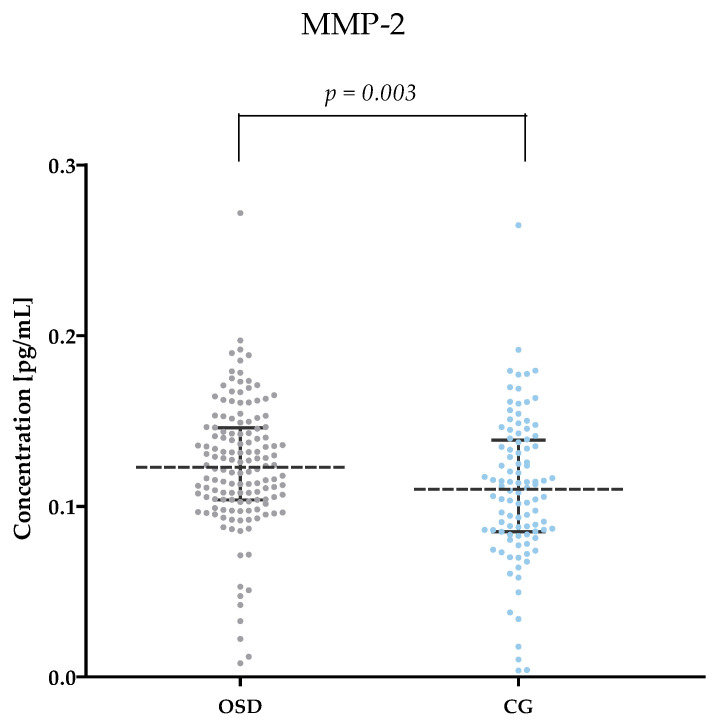
MMP-2 concentrations obtained plasma of subjects with OSD and CG (with marked median and interquartile range).

**Figure 2:**
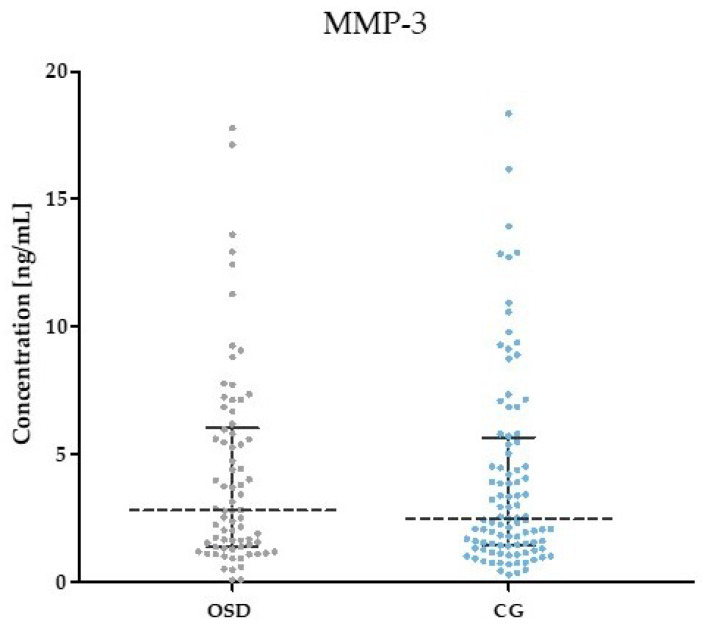
MMP-3 plasma concentrations obtained in the plasma of subjects with OSD and CG (with marked median and interquartile range).

**Figure 3:**
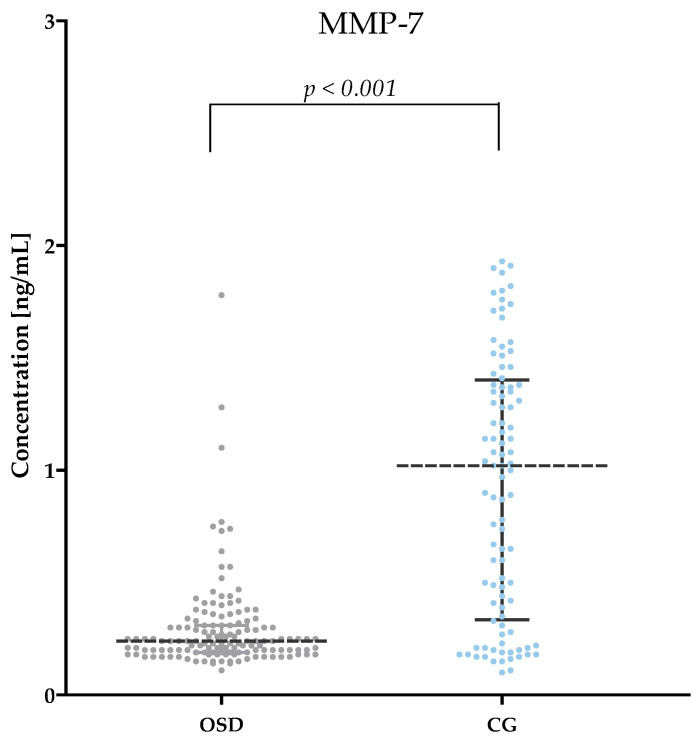
MMP-7 concentrations obtained in the plasma of subjects with OSD and CG (with marked median and interquartile range).

**Figure 4:**
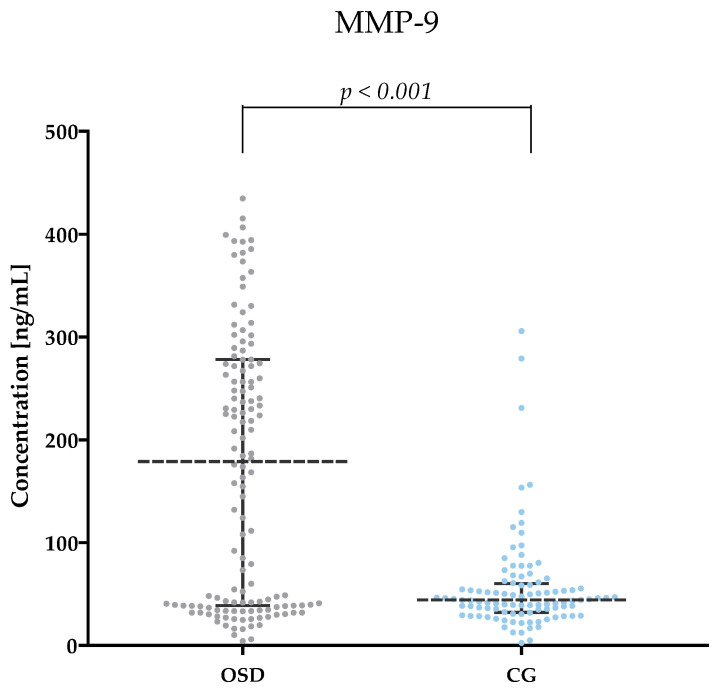
MMP-9 concentrations obtained in the plasma of subjects with OSD and CG (with marked median and interquartile range).

**Figure 5:**
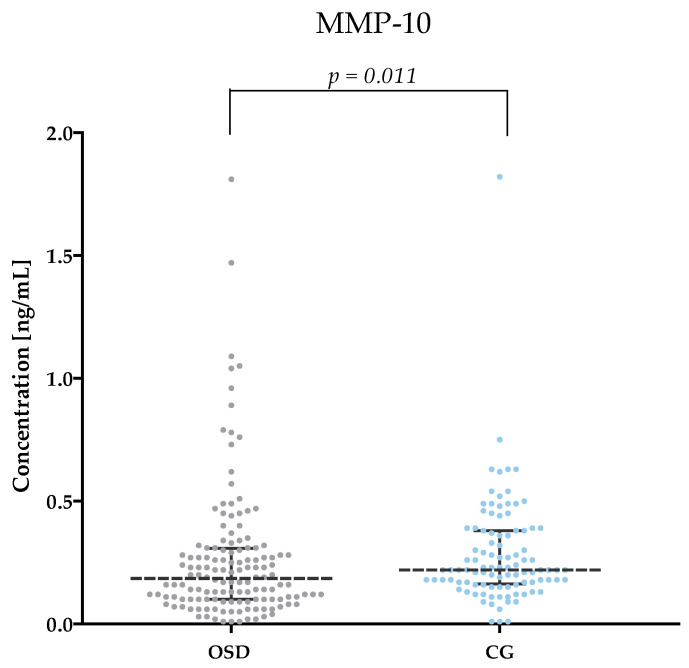
MMP-10 concentrations obtained in the plasma of subjects with OSD and CG (with marked median and interquartile range).

**Figure 6:**
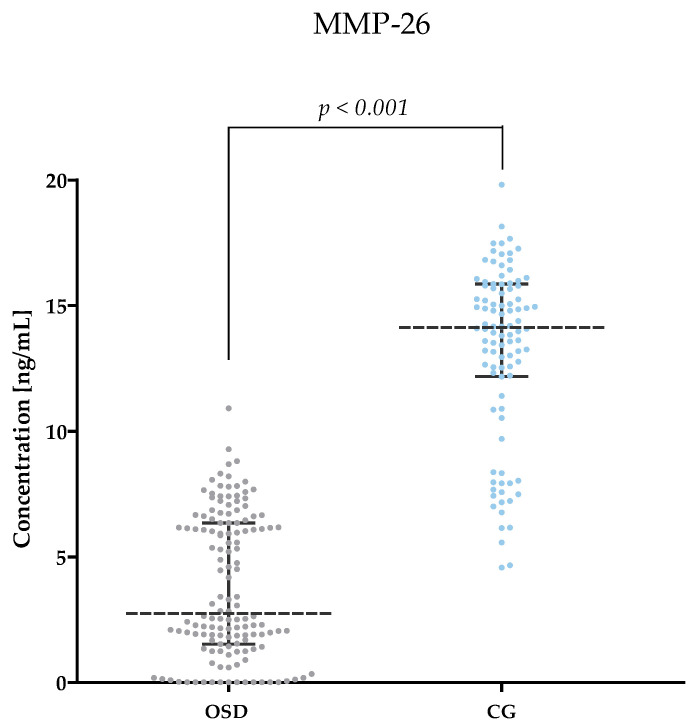
MMP-26 concentrations obtained in the plasma of subjects with OSD and CG (with marked median and interquartile range).

Table 2 and Figure 1, Figure 2, Figure 3, Figure 4, Figure 5 and Figure 6 show the results of the concentrations of the studied parameters: MMP-2, MMP-3, MMP-7, MMP-9, MMP-10 and MMP-26 in the plasma of Osgood–Schlatter (OSD) patients compared to the control group (CG).

### 3.2. MMPs Concentrations in Plasma of OSD Patients and Control Group (CG)

#### 3.2.1. MMP-2 Concentrations in Plasma of OSD Patients and CG Subjects

Statistical analysis showed significantly higher MMP-2 values in OSD patients (median: 0.12 pg/mL) compared to the results obtained in the CG (median: 0.11 pg/mL, *p* = 0.003). The results are shown in Figure 1.

#### 3.2.2. MMP-3 Concentrations in Plasma of OSD Patients and CG Subjects

The results of MMP-3 concentrations in the OSD group (median: 3.11 ng/mL) and the CG (median: 2.48 ng/mL) showed no statistically significant differences (*p* = 0.224). The results are shown in Figure 2.

#### 3.2.3. MMP-7 Concentrations in Plasma of OSD Patients and CG Subjects

Significantly lower serum MMP-7 concentrations were observed of OSD subjects (median: 0.24 ng/mL) compared to those of CG (median: 1.02 ng/mL, *p* < 0.001) (Figure 3).

#### 3.2.4. MMP-9 Concentrations in Plasma of OSD Patients and CG Subjects

Significantly higher MMP-9 values (median: 178.83 ng/mL) were obtained in the plasma of OSD patients compared to the results obtained in the CG (median: 44.20 ng/mL, *p* < 0.001). The results are shown in Figure 4.

#### 3.2.5. MMP-10 Concentrations in Plasma of OSD Patients and CG Subjects

Significantly lower values of MMP-10 levels were obtained in OSD patients (median: 0.19 ng/mL) compared to those obtained in the CG (median: 0.22 ng/mL, *p* = 0.011). The results are shown in Figure 5.

#### 3.2.6. MMP-26 Concentrations in Plasma of OSD Patients and CG Subjects

The lowest MMP-26 concentrations were observed in the OSD group (median: 2.74 ng/mL) compared to the CG (median: 14.13, *p* < 0.001). The results are shown in Figure 6.

### 3.3. Evaluation MMPs Correlation

**Figure 7:**
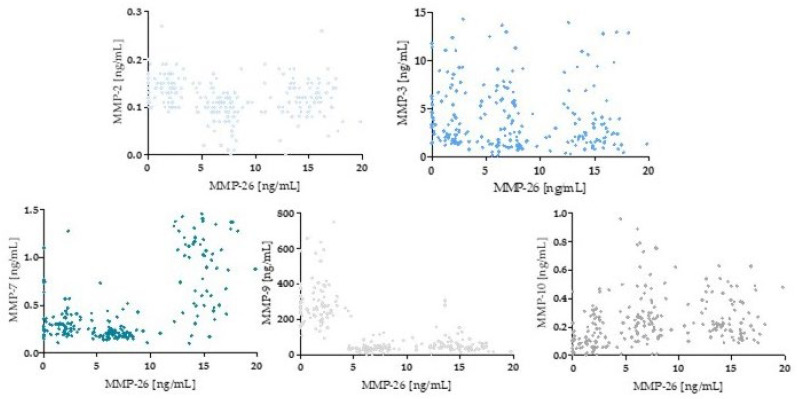
Spearman’s rank correlation test for tested MMPs.

By using Spearman’s non-parametric correlation test, the correlation of the MMPs studied was evaluated. The results are shown in Table 3 and Figure 7.

According to the results of correlation analysis, statistically significant correlations were observed between some MMPs. A statistically significant negative correlation was found between MMP-2 and MMP-26 (r = −0.24, *p* = 0.002); MMP-9 and MMP-10 (r = −0.3 *p* < 0.001); and MMP-9 and MMP-26 (r = −0.58; *p* < 0.001)). In addition, a statistically significant positive correlation was observed between MMP-2 and MMP-9 (r = 0.50, *p* < 0.001) and MMP-7 and MMP-26 (r = 0.46; <0.001).

### 3.4. Diagnostic Usefulness of Tested Parameters: MMP-2, MMP-3, MMP-7, MMP-9, MMP-10 and MMP-26

Evaluation of the diagnostic criteria of the tested MMPs was performed by using the diagnostic accuracy (AC), sensitivity (SE), specificity (SP) and area under curve (AUC). The obtained results are presented in Table 4.

The highest AC value was characterized by MMP-26 reaching the value of this parameter equal to 91%, a slightly lower but still high result was obtained for MMP-7 (83%) and for MMP-2 (68%). The values of MMP-3 and MMP-10 slightly exceeded the value of 50% (MMP-3—55% and MMP-10—59%).

In the case of SE, the highest value was again obtained for MMP-26 (94%); however, MMP-7 (92%) and MMP-2 (90%) obtain similar SE values. SE for the other parameters was much lower and did not exceed the value of 70%—the highest value was obtained for MMP-3 (69%).

**Figure 8:**
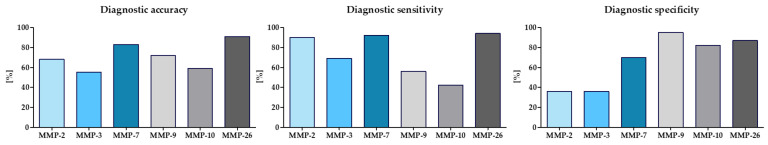
The values of AC, SE and SP for tested MMPs.

Determining the SP, MMP-9 achieves the highest values for this parameter, at 95%. MMP-26 and MMP-10 acquired similar amounts of SP—87% and 82%, respectively. The SP value for MMP-7 showed a value of 70%, while the lowest values of this indicator were acquired for MMP-2 and MMP-3 (both 36%). The values of AC, SE and SP for tested MMPs are shown in Figure 8.

**Figure 9:**
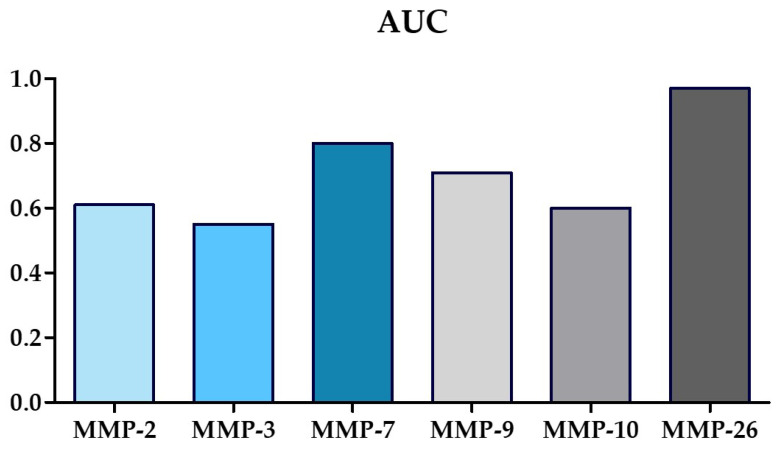
The values of AUC for tested MMPs.

**Figure 10:**
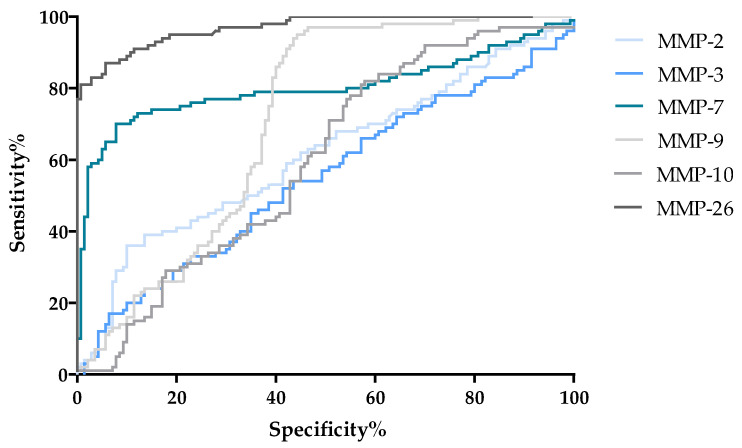
ROC curve for tested parameters in OSD patients.

An AUC value was also determined for the MMPs tested. An AUC value of 0.5 is a borderline of the diagnostic usefulness of the test. The highest AUC values were obtained for MMP-26 (0.97) and MMP-7 (0.80). Slightly lower AUC values were obtained for MMP-9 (0.60). Comparable AUC values were obtained for MMP-2 (0.61) and MMP-10 (0.60). The lowest AUC is characterized by MMP-3 (0.55), but it should be noted that this value exceeds 0.5. The AUC values are shown in Figure 9, while the relationship between SP and SE is represented by the ROC curve shown in Figure 10.

## 4. Discussion

Osgood–Schlatter disease belongs to sterile bone necrosis. It is one of the most common bone pathologies in athletically active children [1,3,22]. It is estimated that OSD can affect up to 20% of athletically active adolescents during their growing years [3]. Treatment of this condition includes immobilization, the use of ice packs and the use of NSAIDs. Attempts are also underway to use leukocyte-rich platelet-rich plasma to treat the condition [3]. OSD mostly affects the male gender; however, girls are increasingly affected by the condition. The strength of bone and tendon structures is influenced by a number of factors related to gender differences, resulting, among other things, from the concentrations of sex hormones. Currently, the treatment of OSD looks the same for both sexes; however, in the future, orthopedic treatment of this condition could be appropriately tailored due to the gender of patients [4]. Although the condition is self-limiting in most cases, some patients require surgical treatment [3,22,23]. Currently, the etiology of this condition is not fully understood; however, scientific reports indicate a significant role of being overweight, tightness in the quadriceps of the thigh and reduced flexibility of the muscles in the posterior group of the thigh [3,23,24]. Knowing the exact etiology and etiopathogenesis of OSD would enable the effective prevention of this condition.

Matrix metalloproteinases (MMPs) are among the enzymes with proteolytic properties, and they physiologically regulate the bone system [12,14]. In addition, some studies indicate that these enzymes are involved in the onset of skeletal diseases such as cervical vertebral degeneration and bone tumors [21]. It is currently unclear whether MMPs are also involved in the pathogenesis of OSD. Therefore, the aim of the present study was to evaluate changes in plasma levels of selected MMPs—MMP-2, MMP-3, MMP-7, MMP-9, MMP-10 and MMP-26—in patients with OSD compared to those with knee pain unrelated to OSD, which will allow us to preliminarily determine the potential of selected MMPs as an additional test to differentiate OSD from other knee pain and their potential involvement in the pathogenesis of OSD.

The first enzyme from the group of MMPs that was assayed in our study is MMP-2. MMP-2 belongs to the gelatinase group. This enzyme is expressed in osteoblast, osteoclasts and osteocytes [25,26]. An increased expression of MMP-2 is associated with an increased osteolysis of bone. This is due to the direct proteolytic action of MMP-2 and indirect mechanisms such as the increased degradation of collagen and osteoid on the bone surface, facilitation of osteoclast recruitment, inhibition of osteoblast differentiation and increased production of pro-osteolytic mediators [27]. In our study, we found that OSD patients (median: 0.12 pg/mL; *p* = 0.003) have slightly higher MMP-2 levels compared to the control group (median: 0.11 pg/mL). Currently, we are the first team to study the plasma concentrations of MMPs in people with OSD; therefore, we have no way to relate our results to the work of other research groups. However, some studies indicate higher concentrations of MMP-2 in the blood of people with bone diseases such as osteosarcoma [28] and osteoporosis [29,30] compared to healthy individuals. These results agree with our data and indicate that osteoskeletal diseases may be associated with increased levels of MMP-2 and that this enzyme may be involved in the pathogenesis of OSD.

MMP-3 belongs to the stromelysin group. This enzyme is weakly expressed in bone cells [31]. Importantly, MMP-3 has been shown to be an important regulator of the action of sex hormones, which is associated with the pathogenesis of osteoporosis [32]. In our study, we found no significant differences between MMP-3 levels in OSD patients (median: 3.11 pg/mL) compared to control subjects (median: 2.48 pg/mL). This is an unexpected result, as previous studies confirm the involvement of MMP-3 in bone degradation—such as in osteoarthritis [33]. It is possible that the changes in MMP-3 activity, however, are only limited to the affected knee and are not associated with changes in blood concentrations of this enzyme. Importantly, in studies conducted by other teams on the potential of MMP-3 as a serum marker in the course of osteoporosis, the concentrations of this enzyme were dependent on the presence of the disease and the menopausal status of patients with known osteoporosis, which may indicate an effect of age on MMP-3 concentrations [34]. In our study, the study group was not gender homogeneous. It is very possible that the concentrations of MMP-3 in female patients, before puberty, will be different from those in male patients and in girls after puberty, but this requires performing studies on two gender groups similar in number.

Another enzyme we determined was MMP-7, which belongs to the matrilysin group. This enzyme is responsible for bone cell differentiation, growth, and importantly, is responsible for regeneration of bone structures [35]. In our study, the plasma levels of MMP-7 were significantly lower in OSD patients (median: 0.24 ng/mL) compared to controls (median: 1.02 ng/mL; *p* < 0.001). Since we are the first research team to establish such a correlation, we are not able to relate our results to studies from other research teams. Such a significant difference in MMP-7 concentrations between the two study groups may indicate a significant role for potential inhibition of MMP-7 activity in OSD formation and indicates the potential of MMP-7 as a negative marker with great potential in an auxiliary test to differentiate OSD from other knee pain. However, this requires further studies, including in vitro studies.

MMP-9, like MMP-2, belongs to the group of gelatinases. The biological properties of MMP-9, among others within the skeletal system, are fairly well understood. MMP-9 expression is found in osteoblasts and osteoclasts [25,26]. This enzyme is responsible for osteoclast/chondroclast recruitment, angiogenesis within bone, and primary ossification [14]. Excessive MMP-9 activity is associated with the development of many skeletal diseases such as bone cancer [28], spinal degeneration [36] and osteoporosis [37]. In the results obtained by our team, we found significantly higher levels of MMP-9 (median: 178.83 ng/mL) compared to control subjects (median: 44.20 ng/mL; *p* < 0.001). This may suggest the involvement of MMP-9 in the pathogenesis of OSD; however, we are unable to relate our results to the work of other teams. A single study by Kushlinsky et al. [19] indicates that patients with osteosarcoma, however, have lower serum MMP-9 levels than healthy individuals. It should be noted, however, that many diseases, not only of the skeletal system, proceed with an increase in blood MMP-9 concentrations [7,10,38]. This may support our assumption of a link between increased MMP-9 activity and OSD formation and the role of this enzyme as a potential differentiation marker in OSD and knee pain.

MMP-10 belongs to the stromelysin group. The functions of this enzyme within bone are poorly understood—some studies indicate a role for MMP-10 in bone development and its increased activity is associated with the degeneration of intervertebral disc structures [39]. In our study, patients with OSD showed slightly lower levels of MMP-10 (median: 0.19 ng/mL) compared to the control group (median: 0.22 ng/mL; *p* = 0.011). The results we obtained are not consistent with the assumption of the potential involvement of MMP-10 in the pathogenesis of OSD. Since there are no studies on MMP-10 concentrations in the plasma or serum of patients with OSD or any other bone disease, we partially compare our results to the work of Aripaka et al. [40], who found higher MMP-10 expression in patients with osteoarthritis and that this expression increased with disease severity. However, serum MMP-10 was not tested. Considering the fact that patients with OSD have lower plasma MMP-10 concentrations and the fact that an important role of this enzyme in the degradation of other bone structures has been confirmed, it is possible that this enzyme is not involved in the pathogenesis of OSD. However, this should be confirmed by studies determining the expression of MMP-10 in the tissue of OSD patients.

The last enzyme studied was MMP-26, which belongs to the matrilysin group. This is the enzyme that is the least understood of all the compounds studied by our team. We are now the first research team to determine the plasma concentrations of MMP-26 not only in OSD, but also in any skeletal disease. MMP-26 degrades fibrinogen, fibronectin, vitronectin and denatured collagen types I–IV. The expression of MMP-26 is found in osteoblasts and intervertebral disc building cells [38]. According to our study, patients with OSD have significantly lower levels of MMP-26 (median: 2.74 ng/mL) compared to the control group (median: 14.13 ng/mL; *p* < 0.001). As with MMP-2 and MMP-9, higher plasma concentrations of this MMP-26 may indicate the diagnostic utility of this enzyme in the differential diagnosis of OSD from knee pain and the potential involvement of this compound in OSD formation.

In order to determine the diagnostic power of the selected MMPs in the study, we used a number of diagnostic tools such as diagnostic accuracy, sensitivity, specificity and test power. Of all the MMPs tested, the best values of diagnostic parameters were obtained for MMP-9 (successively: AC—72%; SE—56%; SP—95%; AUC—71%) and MMP-26 (successively: AC—91%; SE—94%; SP—87%; AUC—97%). Also, for MMP-7, we obtained good values of diagnostic parameters (successively: AC—83%; SE—92%; SP—70%; AUC—80%). Given the good values of the diagnostic parameters, the visible changes in the plasma concentrations of these MMPs, and the scientific reports on their physiological and pathological roles in bone, one can tentatively suggest their involvement in the formation of OSD. The data we have acquired indicate that overactivity of MMP-9 and MMP-26 and reduced activity of MMP-7 may be involved in the pathogenesis of this condition. In addition, the biochemical assessment of the concentrations of these MMPs can be considered as an auxiliary test in the differential diagnosis of OSD from other bone pain not caused by bone necrosis. Knowing the exact pathogenesis of OSD and having additional and non-invasive tests to help differentiate OSD from other diseases will allow the effective prevention of this disease and more effective treatment of patients, especially those requiring surgery. However, this requires further research, including in vitro studies.

Our work also has a number of limitations.

First, the study groups we selected were not equally matched in terms of the number of patients of both sexes. In future studies, we plan to select groups so that the number of patients of both sexes is equal. This will allow us to unequivocally rule out the potential role of gender on the results obtained, which will be important especially for MMP-3.

Secondly, in the study we did not determine the concentrations of metalloproteinase inhibitors such as TIMP-1 (Tissue Inhibitor of Metalloproteinase-1) and TIMP-2 (Tissue Inhibitor of Metalloproteinase-2). We plan to determine these compounds in the next round of the study. This will allow us to identify potential perturbations in the inhibition of MMPs activity in patients with OSD. However, since the present work is a pilot study that only aimed to determine potential changes in MMPs levels in OSD patients, we will analyze TIMPs (Tissue Inhibitor of Metalloproteinases) in the next work.

Third, in the present work, we only present results obtained from patients in the acute phase of OSD. This disease can also have a chronic course; however, due to the limited number of blood samples from patients with this form, they were not included in the study.

In the present study, we found that the concentrations of MMPs are altered during the course of OSD. In the future, we also plan to evaluate changes in MMPs concentrations in patients after OSD treatment, which will allow us to assess their potential in evaluating the effectiveness of treatment for this condition.

In conclusion, we were the first research team to determine the changes in the levels of selected MMPs in patients with OSD. The results obtained in this work indicate the diagnostic potential of MMP-7, MMP-9 and MMP-26 as an additional test in the differential diagnosis of OSD. Our study may also guide further research into this condition in the future, which will reduce the incidence of the disease and the need for surgery in young athletes.

## 5. Future Perspectives

The diagnosis of OSD is based on clinical history and radiological studies. The obtained concentrations of the enzymes tested (MMP-2, MMP-3, MMP-7, MMP-9, MMP-10 and MMP-26) may indicate their role in the pathogenesis of this disease, especially considering that MMPs have been shown to be significantly involved in the pathogenesis of other bone disorders. The above information does not exclude the possibility that a group of these enzymes may represent a group of potential tests to aid in the diagnosis of OSD. In the future, we plan to correlate MMPs concentrations with clinical parameters of OSD, such as the disease severity, duration of symptoms, potential for complications resulting in surgery, and to assess treatment efficacy. To assess treatment efficacy, we want to correlate MMPs concentrations with the most commonly used scales for this purpose, such as the VAS, Tegner, Lysholm and KOOS scales. However, as we have emphasized in the discussion here, we are unable to confirm our theory with absolute certainty as this requires further research, including in vitro studies.

## 6. Conclusions

According to the results we obtained, increased levels of MMP-9 and MMP-26 and lower levels of MMP-7 have the potential to be additional tests in differentiating OSD. The changes in the concentrations of these MMPs may indicate a role for these enzymes in the pathogenesis of OSD.

## Figures and Tables

**Table 1:** Characteristics of patient groups.

Osgood–Schlatter Group (OSD)	
Number of patients	140
Gender of patients	Female—71 Male—69
Median of age	13 years old
**Control Group (CG)**	
Number of patients	100
Gender of patients	Female—38 Male—62
Median of age	13 years old

**Table 2:** Results of plasma concentrations of selected MMPs in OSD patients and CG.

	Osgood–Schlatter Disease		Control Group		*p*
	Median	IQR	Median	IQR	
MMP-2 [pg/mL]	0.12	0.10–0.15	0.11	0.09–0.14	0.003 *
MMP-3 [ng/mL]	3.11	1.66–6.22	2.48	1.43–5.54	0.224
MMP-7 [ng/mL]	0.24	0.19–0.31	1.02	0.34–1.39	<0.001 *
MMP-9 [ng/mL]	178.83	38.67–278.01	44.20	32.28–59.34	<0.001 *
MMP-10 [ng/mL]	0.19	0.10–0.30	0.22	0.17–0.38	0.011 *
MMP-26 [ng/mL]	2.74	1.54–6.35	14.13	12.20–15.85	<0.001 *

IQR- Interquartile range; *—**bold text** indicates statistically significant results.

**Table 3:** Spearman’s rank correlation test for tested MMPs.

Tested Correlations			r	*p*
MMP-2	vs.	MMP-26	−0.24	0.002 *
MMP-2	vs.	MMP-9	0.50	<0.001 *
MMP-7	vs.	MMP-26	0.46	<0.001 *
MMP-9	vs.	MMP-10	−0.3	<0.001 *
MMP-9	vs.	MMP-26	−0.58	<0.001 *

*—**bold text** indicates statistically significant results.

**Table 4:** Values of diagnostic criteria (AC, SE, SP and AUC) obtained for the tested MMPs.

Parameter	AC [%]	SE [%]	SP [%]	AUC
MMP-2	68	90	36	0.61
MMP-3	55	69	36	0.55
MMP-7	83	92	70	0.80
MMP-9	72	56	95	0.71
MMP-10	59	42	82	0.60
MMP-26	91	94	87	0.97

## Data Availability

The raw data supporting the conclusions of this article will be made available by the authors on request. We encourage You to direct inquiries to the corresponding author.

## List of the Abbreviations

AC	Diagnostic Accuracy
AUC	Area Under Curve
CG	Control Group
ECM	Extracellular Matrix
ELISA	Enzyme-Linked Immunosorbent Assay
IQR	Interquartile Range
MMP-10	Matrix Metalloproteinase-10
MMP-2	Matrix Metalloproteinase-2
MMP-26	Matrix Metalloproteinase-26
MMP-3	Matrix Metalloproteinase-3
MMP-7	Matrix Metalloproteinase-7
MMP-9	Matrix Metalloproteinase-9
MMPs	Matrix Metalloproteinases
NSAIDs	Nonsteroidal Anti-Inflammatory Drugs
OSD	Osgood–Schlatter Disease
ROC curve	Receiver Operator Characteristic curve
SE	Sensitivity
SP	Specificity
TIMP-1	Tissue Inhibitor of Metalloproteinase-1
TIMP-2	Tissue Inhibitor of Metalloproteinase-2
TIMPs	Tissue Inhibitor of Metalloproteinase
X-rays	X-radiation
